# Incidence and pathophysiologic mechanisms of stroke in the COVID-19 pandemic: the dilemma

**DOI:** 10.1186/s43168-020-00033-y

**Published:** 2020-09-29

**Authors:** Sherif Mohamed, Seham Abd El-Mohsen, Osama Abo El-Hassan, Azza AbdElHaffez, Nashwa Abd El-Aziz

**Affiliations:** 1 Department of Chest Diseases and Tuberculosis, Faculty of Medicine, Assiut University, Assiut, 71516 Egypt; 2 Department of Neurology, Al-Azhar University, Cairo, Egypt; 3 Department of Pulmonary Medicine, Faculty of Medicine, Cairo University, Cairo, Egypt; 4 Department of Medical Physiology, Faculty of Medicine, Assiut University, Assiut, Egypt; 5 Department of Medical Oncology, South Egypt Cancer Institute, Assiut University, Assiut, Egypt

**Keywords:** COVID-19, Stroke, Pathophysiology, Outcomes

## Abstract

**Background:**

While COVID-19 pandemic affected more than 26 million people worldwide, still, the definite link between COVID-19 and incidence of stroke remains to be re-evaluated.

**Main body:**

Many pathophysiologic and immunologic mechanisms have been implicated in stroke occurring among patients with COVID-19. The COVID-19 pandemic has, in different ways, negative impacts on care of stroke patients worldwide, and still, many challenges are faced by neurologists to improve care of stroke patients during such crisis. In this brief report, we try to discuss these issues.

**Conclusions:**

Although the control of COVID-19 is of crucial importance, at the same time, the management of stroke must not be neglected. Therefore, introducing care for critical conditions such as stroke, and providing strategies to ensure this proceeds, is a priority even at the time of the pandemic.

## Background

The coronavirus infection COVID-19 first presented as an outbreak of atypical pneumonia in Wuhan, China, on December 12, 2019. Since then, it has spread globally to infect over 26 million people [1]. The definite link between COVID-19 and the prevalence of stroke remains to be determined. Many pathophysiologic and immunologic mechanisms have been implicated in stroke occurring among patients with COVID-19. In this mini-review, we will discuss the links between COVID-19 and stroke.

## Main text

### Incidence of stroke at the time of COVID-19 pandemic: increased or decreased?

Despite the worldwide spread of COVID-19, the true relationship between it and the incidence of stroke remains to be elucidated. It has been suggested that COVID-19 infection, by itself, may lead to stroke. In an interesting study from Wuhan, China, 36.4% of COVID-19 patients had symptoms of neurological affection, which were more encountered in those with severe disease [2]. Among those neurological disorders, stroke was a complication of COVID-19 infection in 5.9% of patients. Characteristically, patients with stroke were older, had more severe pneumonia, and more cardiovascular comorbidities [2]. In comparison to influenza virus, a recent study has reported that 1.5% of patients with emergency department visits or hospitalizations with COVID-19 experienced ischemic stroke, a rate 7.5-fold higher than in patients with influenza [3]. Mechanisms by which COVID-19 might increase the risk of stroke have been addressed. These mechanisms may include hypercoagulability status due to critical illness and intracardiac thrombosis and pulmonary embolism from SARS-CoV-2-related coagulopathy. As the obligate receptor for the virus, human angiotensin-converting enzyme (ACE-2) is expressed in epithelial cells throughout the body, including in the central nervous system (CNS); this raises the probability of a direct role in viral infection [4, 5]. On the contrary, at the onset of the COVID-19 era, there was an apparent reduction in the number of stroke cases in many parts of the world. Reports pulled out from multiple countries showed a marked drop in the number of stroke-related hospitalizations [4]. In a retrospective cohort study of consecutive patients with ischemic stroke who were hospitalized between March 15, 2020, and April 19, 2020, within a major health system in New York, Yaghi and coworkers found that, out of 3556 hospitalized patients with diagnosis of COVID-19 infection, 32 patients (0.9%) had imaging-proven ischemic stroke. Cryptogenic stroke was more common in patients with COVID-19 (65.6%) as compared to contemporary controls (30.4%, *p* = 0.003) and historical controls (25.0%, *p* < 0.001) [6]. Many explanations for these reduced numbers of stroke cases were considered. Patients with milder stroke may have reduced rates of admissions; some patients have fears of getting infected if they were referred to the hospital during times of the pandemic. In a recent report from Italy, other mechanisms were claimed [7]. First, IL-6 plays a controversial role in stroke. While high levels of serum markers for thrombosis and inflammation have been reported in COVID-19-affected patients, as well as increased levels of inflammatory cytokines (interleukin [IL]-2R, IL-6, and tumor necrosis factor-α) [8]. Indeed, there were debates if high IL-6 levels have a negative influence on the volume of brain infarct and/or long-term outcomes [7]. On the contrary, there was supportive evidence that IL-6 may have a protective effect and helps in the improvement of the angiogenesis process in patients with ischemic stroke [7, 8].

Second, a possible explanation is related to the observation that most COVID-19 patients have thrombocytopenia [9]. Some authors wondered if thrombocytopenia was related to the reduction in the incidence of large vessel occlusion (LVO) strokes. Third, the burden of chronic persistent infections rather than one single current pandemic could be associated with risk for cerebrovascular disease [7]. Another explanation may come from the observation that air pollution is associated with an increased risk of cardiovascular disease; we had seen a strikingly reduced air pollution during the pandemic secondary to lockdown; this phenomenon could have a protective effect against stroke [4].

### Is stroke a risk factor for COVID-19 or is COVID-19 a risk factor for stroke?

From our experience, it was observed that the presence of cerebrovascular disease (CVD) in patients with SARS-CoV-1 or MERS-CoV was associated with worse outcomes. Still, we do not know whether CVD predicts outcomes of patients with SARS-CoV-2 or not. A pooled analysis has showed an ~ 2.5-fold increase in odds of severe COVID-19 in patients with a history of CVD, but there was no association with mortality [10]. On the other hand, in patients with stroke, the presence of COVID-19 infection itself was thought to be a potential factor in the genesis or worsening of cerebrovascular stroke. The virus causing COVID-19 can enter the CNS through two different pathways: retrograde neuronal diffusion and via the blood-brain barrier. Also, the spread of COVID-19 through the cribriform plaque of the ethmoid bone can lead to brain involvement. This could happen during the initial phase or at subsequent infection. Notably, the presence of ACE-2 receptors on both neuronal and capillary endothelial cells could give rise to the subsequent spread and damage to the cerebral nervous system, characteristically without substantial inflammatory load [4, 5].

Oxley et al. [11] reported five cases of large vessel stroke over 14 days in COVID-19 patients aged under 50 years. Remarkably, all cases had either no or mild COVID-19 symptoms. Interestingly, this observation represents a sevenfold increase in what would normally be noticed.

### Impacts of the COVID-19 pandemic on the care of stroke patients and challenges to the neurologist

The COVID-19 pandemic has both direct and indirect major implications for stroke care. A minority of countries did manage to maintain a full range of coverage for stroke services. However, the other majority has experienced significant service shortage and/or reorganization. The latter faced 2 problems. First, reallocation of neurology care beds including those of the ICU to manage COVID-19 patients necessitated moving of stroke units to a less optimal situation. Second, the crisis needed redeployment of stroke physicians, nurses, and other stroke facilities to look after COVID-19 patients [12]. At the best services, intravenous thrombolysis is under threat because of pressures and delays resulted from managing potentially infected patients. This has resulted in missing the therapeutic window at the worst stroke patients. Adding to this dilemma, there were delays in hospitalizations for stroke, or even patients preferring not to be hospitalized at all. The impact on developing countries was eventually worse. Many have not only much less-developed stroke services but also less-developed usual care for managing COVID-19 patients, including the major challenge of shortage of ventilators in the intensive care units [13].

### Potential solutions

Best practice guidelines had to offer guidelines for the best way(s) to manage stroke in the context of the current pandemic while keeping in mind the safety of the healthcare workers. Telemedicine was found to offer many solutions during this pandemic. Stroke has led the way in telemedicine for proper assessment for thrombolysis, which continues to be of crucial importance for stroke care particularly in rural settings. Utilizing telemedicine had avoided the use of excessive protection, allowed a reasonable stroke evaluation, and reduced the risk of exposure for the stroke-managing team [4, 7, 11].

## Conclusions

Although the control of COVID-19 is of crucial importance, at the same time, the management of stroke must not be neglected. Therefore, introducing care for critical conditions such as stroke, and providing strategies to ensure this proceeds, is a priority even at the time of the pandemic.

## Data Availability

Not applicable

## Abbreviations

ACE-2: Angiotensin-converting enzyme
CNS: Central nervous system
COVID-19: Coronavirus disease 2019
CVD: Cerebrovascular disease
MERS: Middle East respiratory syndrome
LVO: Large vessel occlusion
SARS: Severe acute respiratory syndrome
